# A case of disseminated perforating necrobiosis lipoidica

**DOI:** 10.1002/ccr3.2766

**Published:** 2020-03-27

**Authors:** Niccolò Gori, Alessandro Di Stefani, Erika Valentina De Luca, Ketty Peris

**Affiliations:** ^1^ Institute of Dermatology Catholic University of Rome Rome Italy; ^2^ Fondazione Policlinico Universitario A. Gemelli IRCCS Rome Italy

**Keywords:** diabetes mellitus, granulomatous skin disease, necrobiosis lipoidica, perforating dermatosis

## Abstract

Perforating necrobiosis lipoidica (PNL) is a granulomatous inflammatory skin disease that usually occurs in patients with diabetic. We present a case of a female patient affected by a disseminated form of necrobiosis lipoidica.

## Graphical abstract

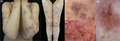

## 1 CLINICAL CASE

**Figure 1:**
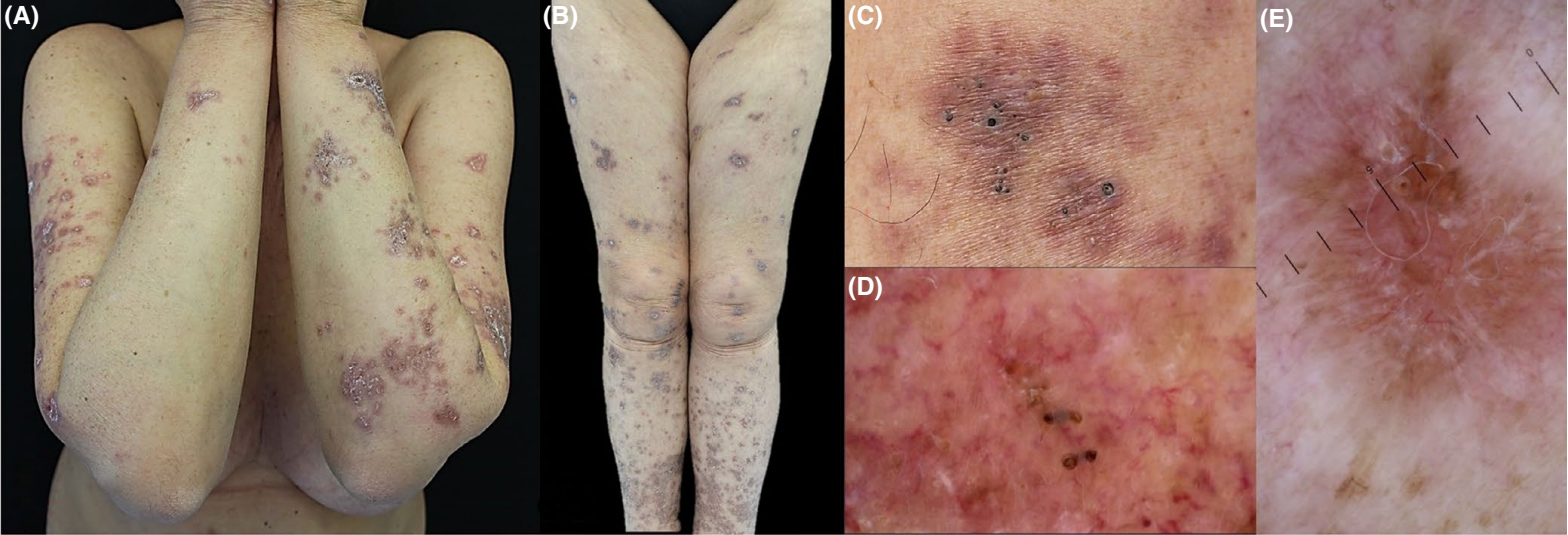
Clinical and dermatoscopic images. A‐B, Red papules and plaques, with different size and shape, characterized by scaly, atrophic areas, and keratin plugs, located on upper and lower extremities. C, Detail of follicular dilated ostia with hyperkeratotic plugs. D‐E, Dermoscopy showing structures similar to comedo‐like openings, irregular linear vessels, and hyperkeratosis on a whitish/yellow background

**Figure 2:**
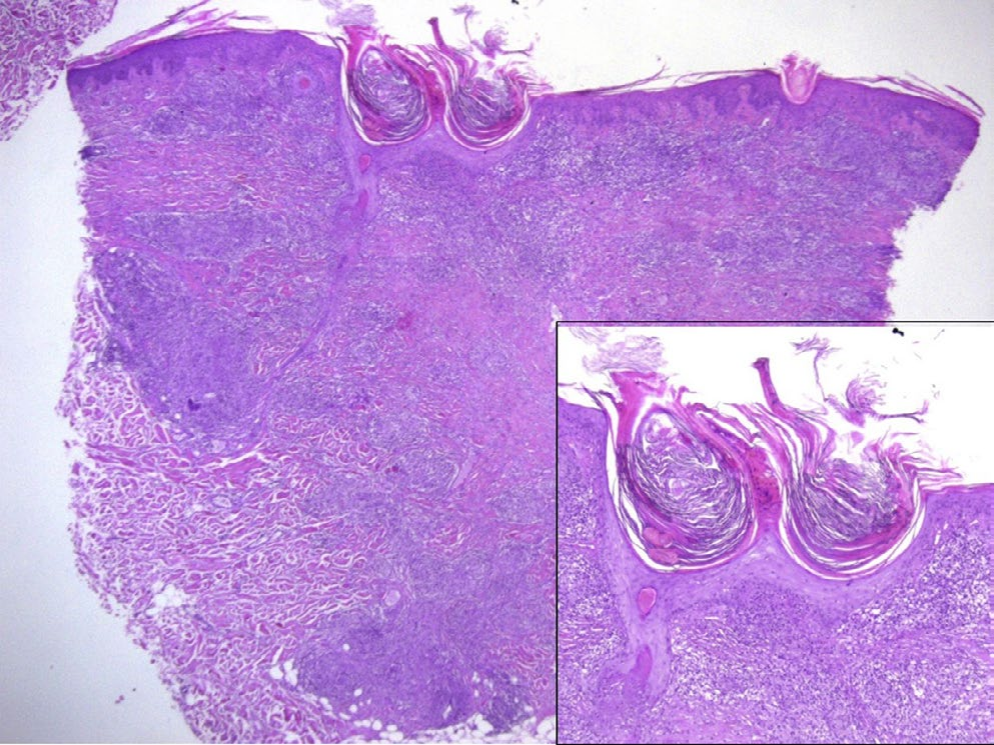
Histopathological images. Granulomatous dermatitis involving the entire derma, with areas of collagen degeneration. Inset transfollicular extrusion of degenerated material. (Hematoxylin and eosin stain, original magnification ×40 and ×100 [inset])

A 60‐year‐old woman presented with a 2‐year history of skin lesions located on the trunk and extremities. Medical history showed diabetes mellitus type 2 and hypertension treated with metformin and bisoprolol, respectively. Physical examination showed red papules and plaques, with different size and shape characterized by scaly, atrophic areas, and keratin plugs, distributed mainly on the upper and lower extremities, and buttocks (Figure 1A‐C). Dermatoscopy examination evidenced a whitish/yellow background with irregular linear vessels, hyperkeratosis, and follicular structures resembling comedo‐like openings (Figure 1D‐E). Histological examination revealed a granulomatous dermatitis involving the entire derma, with areas of collagen degeneration (necrobiosis) and the presence of transfollicular extrusion of amorphous material (Figure 2B). On the basis of clinicopathologic findings, the diagnosis of disseminated perforating necrobiosis lipoidica was made. Treatment with metilprednisolone 30 mg/d for three months resulted in partial clinical improvement.

## 2 DISCUSSION

Necrobiosis lipoidica (NL) is a chronic granulomatous skin disease frequently occurring in patients with diabetes mellitus.^1^ NL typically affects lower legs of middle‐age woman, manifesting as erythematous papules, coalescing in plaques, characterized by an erythematous rim and a yellow‐brown atrophic and telangectatic center.^2^ Pathologically, the lesions reveal degeneration of collagen and a granulomatous inflammation throughout the dermis with possible involvement of subcutaneous tissue.^2^ Parra el al.^3^ first described three patients with NL associated with transfollicular elimination of degenerated collagen. This variant, named “perforating necrobiosis lipoidica” (PNL), has been classified within perforating dermatosis, a group of skin diseases characterized by the extrusion of degenerated connective substances (collagen, elastin, and keratin) through the epidermidis and/or follicular units. PNL is clinically distinguished by numerous keratin plugs and hyperkeratotic papules/plaques, corresponding to areas where degenerated connective tissue is eliminated through the follicular units and epidermidis.^4^ Dermatoscopy can be an useful tool in the diagnosis of this rare variant helping to visualize typical features like a whitish/yellow background and irregular linear vessels, which are suggestive of a granulomatous disease.^5^, ^6^ Furthermore, identification of structures similar to comedo‐like openings, representing areas of degenerated connective tissue, arise the suspect of a perforating cutaneous disorder.

Approximately 90% of cases of PNL described in literature are associated with diabetes mellitus, although unrelated to glucose control.^7^ Lesions are usually located on the lower extremities, less frequently on the upper extremities and trunk. The presence of disseminated lesions, as in our patient, was described only in one case report describing a 42‐year‐old female patient with a 7‐year history of diabetes on insulin therapy, affected by PNL located on upper and lower extremities.^7^

Pathogenesis of perforating disorders is still matter of discussion. Some authors hypothesized that an abnormal keratinization process, occurring in the basal layer of epidermis instead of the upper layer, might result in an inflammatory response against keratin, with subsequent alteration of connective tissue and its extrusion together with keratin and necrotic cells through epidermidis.^8^

Furthermore, the high prevalence of perforating disorders in diabetes mellitus led other authors to suggest a key role of advanced glycosylation end products and oxidized low‐density lipoproteins in provoking host inflammatory response against connective tissue.^9^ The disease has a chronic course with a tendency toward scarring and ulceration.^7^

Treatment is often unsuccessful. Topical and intralesional corticosteroids are preferred in mild and localized cases, while systemic therapy (corticosteroids, cyclosporine, ticlopidina, nicotinamide, and clofazimine) can be used in severe and refractory cases.^10^

In conclusion, the case discussed herein is a rare variant of NL with few cases described in literature.^7^

Clinical diagnosis of PNL can be reached only through an accurate clinical‐pathological correlation. Dermoscopy can be an useful tool helping to identify criteria suggestive for a granulomatous perforating disorder.

## CONFLICT OF INTEREST

None to declare.

## AUTHOR CONTRIBUTIONS

NG: wrote the manuscript. AD and ED: revised the manuscript. KP: supervised the final draft.
